# Does urbanicity modify the relationship between a polygenic risk score for depression and mental health symptoms? Cross-sectional evidence from the observational HUNT Study in Norway

**DOI:** 10.1136/jech-2020-214256

**Published:** 2020-06-24

**Authors:** Erik Reidar Sund, Frank J van Lenthe, Mauricio Avendano, Parminder Raina, Steinar Krokstad

**Affiliations:** 1 HUNT Research Centre, Department of Public Health and Nursing, Faculty of Medicine and Health Sciences, Norwegian University of Science and Technology, Levanger, Norway; 2 Faculty of Nursing and Health Sciences, Nord University, Levanger, Norway; 3 Levanger Hospital, Nord-Trøndelag Hospital Trust, Levanger, Norway; 4 Department of Public Health, Erasmus MC, Rotterdam, Netherlands; 5 Department of Human Geography and Spatial Planning, Utrecht University, Utrecht, Netherlands; 6 Department of GLobal Health and Social Medicine, King’s College London School of Social Science and Public Policy, London, UK; 7 Department of Social and Behavioral Sciences, Harvard T.H. Chan School of Public Health, Harvard University, Cambridge, Massachusetts, USA; 8 Department of Health Research Methods, Evidence, and Impact, McMaster University, Hamilton, Canada; 9 McMaster Institute for Research on Aging, McMaster University, Hamilton, Canada; 10 Labarge Centre for Mobility in Aging, McMaster University, Hamilton, Canada

**Keywords:** EPIDEMIOLOGY, MODELLING, Health inequalities, ECONOMICS, EMPLOYMENT, INTERNATIONAL HEALTH, SOCIAL EPIDEMIOLOGY, AGEING, BIOSTATISTICS, ELDERLY, Epidemiological methods, DISABILITY, HEALTH SERVICES, MENTAL HEALTH

## Abstract

**Background:**

Research suggests that genetic predisposition for common mental disorders may be moderated by the environment. This study examines whether a polygenic risk score (PRS) for depression is moderated by the level of residential area urbanicity using five symptoms of poor mental health as outcomes.

**Methods:**

The study sample consisted of 41 198 participants from the 2006–2008 wave of the Norwegian HUNT study. We created a weighted PRS for depression based on 99 variants identified in a recent genome -wide association study. Participants were classified into urban or rural place of residence based on wards that correspond to neighbourhoods. Mixed effects logistic regression models with participants nested in 477 neighbourhoods were specified.

**Results:**

A SD increase in PRS for depression was associated with a small but statistically significant increase in the odds of anxiety, comorbid anxiety and depression and mental distress. Associations for depression were weaker and not statistically significant. Compared with urban residents, rural resident had higher odds for reporting poor mental health. Genetic propensity for depression was higher for residents of urban than rural areas, suggesting gene–environment correlation. There was no sign of effect modification between genetic propensity and urbanicity for depression, anxiety, comorbid anxiety and depression, or mental distress.

**Conclusion:**

The PRS predicted small but significant odds of anxiety, comorbid anxiety and depression and mental distress, but we found no support for a differential effect of genetic propensity in urban and rural neighbourhoods for any of the outcomes.

## INTRODUCTION

Common mental disorders are a leading contributor to morbidity and disability and represent a substantial public health problem worldwide.^1^ Both depressive disorders, characterised by sustained symptoms of sadness, low energy and sleep disturbances, as well as anxiety disorders, defined by excess worry, hyperarousal and fear, are highly prevalent^2 3^ and they show a high degree of comorbidity.^4^ The risk of common mental disorders varies by age, sex, socioeconomic status and has also been found to vary geographically.^2 5^

The aetiology of both depression and anxiety is complex, but likely determined by genetic, social and environmental factors in a complex interplay. Discoveries from genome-wide association studies (GWAS) suggest that mental health disorders are highly polygenic, that is, they are influenced by hundreds or thousands of genetic variants each having a small effect,^6^ but overall determining an individuals’ genetic predisposition. On their own, however, genetic factors are unlikely to explain a large share of variation in mental health disorders, which are also strongly influenced by the environment. One important environmental factor is captured by urbanicity, which refers to the impact of living in urban areas at a given point in time, and the presence of conditions that are more prevalent than in non-urban areas.^7^ This may confer both an *urban penalty,* for example, by increasing exposure to air pollution or violence, or an *urban advantage*, conferred by higher access to services, cultural activities or social networks. Individuals living in rural areas will generally experience a different environment, typically less stressful, less noise and with much less air pollution. A recent review found conflicting evidence for urban–rural variation prevalent for common mental disorders.^8^

The recognition that both genes (‘nature’) and environments (‘nurture’) contribute to the aetiology of psychiatric disorders has motivated the study of gene–environment interactions (GxE). GxE studies examine to what extent genetic propensity modifies the association between environmental factors and mental health, or conversely, how environmental factors modify associations between genes and mental health. Conceptually, this line of inquiry builds on the diathesis–stress model that posits that genetic propensity (diathesis) interacts, for example, with stressful life events (SLE) to give rise to adverse mental health outcomes. According to this model, genes may exacerbate or buffer the effects of stressful environments. Previous studies on depression rooted in the diastasis–stress model and using polygenic risk scores (PRS) have shown inconsistent results.^9–^ ^11^ A recent test of the diathesis–stress model on depression using PRS and SLE found a significant diathesis–stress interaction,^12^ but these results are yet to be reproduced. The majority of GxE studies adhere to the diathesis–stress model, but alternatives like the differential susceptibility model exist.^13^ According to this model, individuals vary in their susceptibility to both positive and negative environmental influences rather than claiming that specific genotypes are good or bad.

In this study, we aim to assess the hypothesis that the urban environment modifies the relationship between genes and mental health disorders. The majority of GxE studies within the domain of mental health have used the term ‘environment’ to refer to individual-level factors such as behaviour or major life events,^14^ while no studies have examined the interaction between genes and the wider physical and social environment. Our study is based on the Nord-Trøndelag Health study (HUNT), a large general population-based study with substantial variation in level of urbanicity and with detailed genetic data, that enables assessing differential effects of genetic propensity on five mental health outcomes by level of urbanicity.

## METHODS

### Data material

Data from the third wave of the Nord-Trøndelag Health study (HUNT3) was used.^15^ The total population above 19 years in the Nord-Trøndelag county were invited (N=93 860) of which 50 802 participated, yielding a response of 54%. The data include questionnaire information on health, lifestyle, drug treatment and relational issues like family situation. Clinical measurement data and blood samples were collected at screening stations established on several locations (N=23) in the county. Due to the administration of the two main questionnaires (the first sent by mail and brought to the screening station and the second received at the screening station and mailed afterwards), a lower number of respondents had answered the second questionnaire that contained questions on mental health (N=41 198). A study among non-respondents conducted after HUNT3 found that non-participants were more likely to have lower socioeconomic status, higher mortality and a higher prevalence of chronic diseases.^16^ The regional committee for medical research ethics approved the study and all participants provided written consent.

### Outcome measures

Two different measurement instruments for mental health were used in HUNT3: The Hospital Anxiety and Depression Scale (HADS) measures symptoms of anxiety and depression and consists of 14 questions where seven relates to anxiety (HADS-A) and seven to depression (HADS-D). Each subscale ranges from 0 to 21 and a score of ≥8 has been found to be the optimal cut-off with a sensitivity and specificity of ca. 0.8.^17^ Comorbid anxiety and depression were also constructed based on these cut-offs. For the depression subscale, we additionally chose a cut-off of 11 (≥11) to indicate a more severe symptom load.^18^

The Mental Health Index (MHI) consists of seven items with the purpose of measuring mental distress and was calculated by the HUNT databank. The initial question was as follows: Have you in the last two weeks, felt nervous and unsettled, troubled by anxiety, secure and calm, irritable, happy and optimistic, sad/depressed, lonely? Each item had four answer categories ranging from ‘no’ to ‘very’ which were given values from 1 to 4. The average on these seven items were calculated and ranges from 1 to 4. An average MHI ≥2.15 was used to define a high mental distress symptom load that has previously been shown to be a reasonable cut-off compared with HSCL-10 and HADS.^19^

### Main exposure measures

#### Genetics

The PRS is based on genotyping of all participants providing biological samples including DNA. The genotyping was done with one of three different Illumina HumanCoreExome arrays (HumanCoreExome12 v1.0, HumanCoreExome12 v1.1 and UM HUNT Biobank v1.0) as previously described.^20^ Details about genotype quality control and imputation are provided in the online supplementary materials.

A weighted PRS was created based on a recent genome-wide meta-analysis which identified 102 genome-wide significant variants (p<5×10^−8^) associated with depression.^21^ The phenotypes in the GWAS were a mixture of self-reported mental health and clinically derived information (see online supplementary materials). Ninety-nine variants were available in HUNT, and based on the summary statistics (effect allele and effect size), we calculated, for each individual, a PRS value as the weighted sum of risk alleles with the weight being the effect sizes in the GWAS.^6 22^ Finally, the PRS was standardised to a mean of 0 and a SD of 1 to aid interpretation. Prior to the PRS construction, we recoded and ensured that all single-nucleotide polymorphisms in HUNT had the same effect allele as reported in the genome-wide meta-analysis.^21^

10.1136/jech-2020-214256.supp1 Supplementary data

#### Urbanicity

Urbanicity was based on secondary ecological data describing features of 477 geographical wards from the Norwegian Mapping Authority. We had information on place of residence in these wards (average population size=79) for all participants. Wards were classified as rural if no residential houses within a ward were closer than 50 metres apart, whereas the remainder were classified as urban. This classification is based on Statistics Norway’s definition of an urban area. An alternative three-group classification of urbanicity was also constructed. Rural wards were like the previous classification. Wards where the proportion of inhabitants living close (less than 50 metres apart) was larger than the rural category and less than 20% were classified as ‘semi-urban’. The remainder living in wards where more than 20% were living close were classified as ‘urban’.

### Covariates

All models controlled for age (entered as a restricted cubic spline (RCS) with 4 knots), sex and five ancestry-informative principal components (PCs), which account for population stratification.

### Statistical analysis

Mixed effect logistic regression models were used to account for the data structure with individuals nested in 477 wards.^23^ First, we regressed each outcome on the PRS adjusting for age (RCS), sex and the first five ancestry-informative PCs (model 1). Second, we added urbanicity (model 2), and third, we expanded the models by adding an interaction term between the PRS and urbanicity (model 3). Fixed effects are reported as ORs with 95% CIs and random effects as variances on the log-odds scale.

Effects from interaction terms in non-linear models are scale-dependent and the current advice is to report interactions on both the additive (as differences) and multiplicative scale (as ratios).^24^ While interactions on the multiplicative scale in non-linear models are readily available, additive interactions require some extra calculations and here we followed recommendations from recent methodological literature.^25^ Specifically, from model 2 we calculated the marginal effects of the PRS for rural and urban individuals, respectively. These represent the average marginal effect of the PRS on the outcome, which is similar to a test for simple slopes for urban and rural individuals. We subsequently tested if these average marginal effects were different between urban and rural individuals using p<0.05 as the threshold for statistical significance. In an additional test for additive interactions, we also specified linear probability models. Given that interactions can be hard to interpret, we visualised the predictions according to the urban–rural place of residence and the PRS for one of the outcomes (HADS-D8).

We also specified a model to investigate gene-environment correlations (rGE) by regressing urbanicity on the PRS adjusting for age, sex and ancestry. Checking for rGE is important because what appears as interactions may in fact be correlations, that is, the level of genetic propensities may be different in urban and rural wards. We performed a complete case analysis excluding participants with missing values. Data management and statistical modelling were performed in Stata v.15.^26^

## RESULTS

Table 1 shows the descriptive characteristics of the sample. Their mean age was 54.4 years, there were more women (56%) than men, and most participants lived in urban neighbourhoods (70%). There were between 4% and 7.4% missing on the outcomes. Symptoms of anxiety were the most prevalent condition (13.6%), while symptoms of severe depression (HADS-D cut-off 11) were the least prevalent condition (2.2%).

**Table 1:** Descriptive characteristics of the HUNT 3 population in 2006–2008 (N=41 198)

		N	M (SD)/%
Age	Continuous	41 198	54.4 (15.7)
Polygenic risk score (z-score)			
	Continuous	39 782	0 (1)
	*Missing*	*1416*	*3.4*
Sex			
	Women	23 138	56.2
	Men	18 060	43.8
Urbanicity (2 groups)			
	Rural	12 106	29.4
	Urban	28 615	69.5
	*Missing*	*477*	*1.2*
Urbanicity (3 groups)			
	Rural	12 106	29.4
	Semiurban	16 279	39.5
	Urban	12 336	29.9
	*Missing*	*477*	*1.2*
Symptoms			
Depression (HADS-D≥8)			
	No	35 794	86.9
	Yes	3772	9.2
	*Missing*	*1632*	*4.0*
Depression (HADS-D≥11)			
	No	38 675	93.9
	Yes	891	2.2
	*Missing*	*1632*	*4.0*
Anxiety (HADS-A≥8)			
	No	33 677	81.7
	Yes	5594	13.6
	*Missing*	*1927*	*4.7*
HADS A&D≥8			
	No	38 083	92.4
	Yes	1882	4.6
	*Missing*	*1233*	*3.0*
Mental distress (MHI≥2.15)			
	No	35 471	86.1
	Yes	2682	6.5
	*Missing*	*3045*	*7.4*

HADS, Hospital Anxiety and Depression Scale; MHI, Mental Health Index.

Model 1 in table 2 shows the main effects of the PRS on the five mental health outcomes adjusted for age, sex and ancestry. A SD increase in PRS was associated with a significant 1.08 (95% CI 1.05 to 1.12) increased odds of moderate-to-severe anxiety (HADS-A 8), a 1.05 (95% CI 1.00 to 1.10) increased odds of comorbid A&D and a 1.08 (95% CI 1.04 to 1.12) increased odds of mental distress. By contrast, associations were not significant for moderate-to-severe depressive symptoms (HADS-D8) (1.03, 95% CI 0.99 to 1.06) and severe depression (HADS-D11) (1.05, 95% CI 0.98 to 1.12).

**Table 2:** Associations^§^ between a polygenic risk score for depression and five mental health outcomes.

	HADS-D (≥8)		HADS-D (≥11)		HADS-A (≥8)		Comorbid A&D		Mental health score	
	OR	95% CI	OR	95% CI	OR	95% CI	OR	95% CI	OR	95% CI
Model 1										
*Fixed effects*										
PRS (z-score)	1.03	(0.99 to 1.06)	1.05	(0.98 to 1.12)	**1.08**	**(1.05 to 1.12)**	**1.05**	**(1.00 to 1.10)**	**1.08**	**(1.04 to 1.12)**
Sex										
Women	1	ref.	1	ref.	1	ref.	1	ref.	1	ref.
Men	**1.20**	**(1.12 to 1.28)**	1.14	(0.99 to 1.30)	**0.55**	**(0.52 to 0.59)**	**0.78**	**(0.70 to 0.86)**	**0.81**	**(0.75 to 0.88)**
*Random effects*										
Variance (SE)	0.070	(0.015)	0.097	(0.039)	0.036	(0.009)	0.068	(0.022)	0.081	(0.019)
Model 2										
*Fixed effects*										
PRS (z-score)	1.03	(0.99 to 1.06)	1.05	(0.98 to 1.12)	**1.08**	**(1.05 to 1.12)**	**1.05**	**(1.00 to 1.10)**	**1.08**	**(1.04 to 1.12)**
Sex										
Women	1	ref.	1	ref.	1	ref.	1	ref.	1	ref.
Men	**1.19**	**(1.11 to 1.28)**	1.13	(0.98 to 1.30)	**0.55**	**(0.52 to 0.59)**	**0.77**	**(0.70 to 0.85)**	**0.81**	**(0.75 to 0.88)**
Urbanicity										
Urban	1	ref.	1	ref.	1	ref.	1	ref.	1	ref.
Rural	**1.18**	**(1.08 to 1.29)**	**1.33**	**(1.14 to 1.56)**	**1.08**	**(1.00 to 1.16)**	**1.16**	**(1.03 to 1.30)**	1.01	(0.91 to 1.12)
*Random effects*										
Variance (SE)	0.060	(0.014)	0.080	(0.035)	0.034	(0.009)	0.060	(0.022)	0.081	(0.019)
Model 3										
*Fixed effects*										
PRS (z-score)	**1.04**	**(1.00 to 1.09)**	**1.09**	**(1.00 to 1.18)**	**1.08**	**(1.04 to 1.12)**	**1.05**	**(1.00 to 1.12)**	**1.08**	**(1.03 to 1.14)**
Sex										
Women	1	ref.	1	ref.	1	ref.	1	ref.	1	ref.
Men	**1.19**	**(1.11 to 1.28)**	1.13	(0.99 to 1.30)	**0.55**	**(0.52 to 0.59)**	**0.77**	**(0.70 to 0.85)**	**0.81**	**(0.75 to 0.88)**
Urbanicity										
Urban	1	ref.	1	ref.	1	ref.	1	ref.	1	ref.
Rural	**1.18**	**(1.08 to 1.29)**	**1.34**	**(1.15 to 1.56)**	**1.08**	**(1.00 to 1.16)**	**1.16**	**(1.03 to 1.30)**	1.01	(0.91 to 1.12)
PRS*Rural	0.96	(0.89 to 1.03)	0.91	(0.79 to 1.05)	1.00	(0.94 to 1.07)	0.98	(0.89 to 1.09)	0.99	(0.90 to 1.08)
*Random effects*										
Variance (SE)	0.060	(0.014)	0.080	(0.035)	0.034	(0.009)	0.060	(0.022)	0.081	(0.019)

§Adjusted for age (restricted cubic splines with 4 knots) and first five principal components. Estimates in bold significant at p<0.05. HADS, Hospital Anxiety and Depression Scale; PRS, polygenic risk score. ref.=reference category

**Figure 1:**
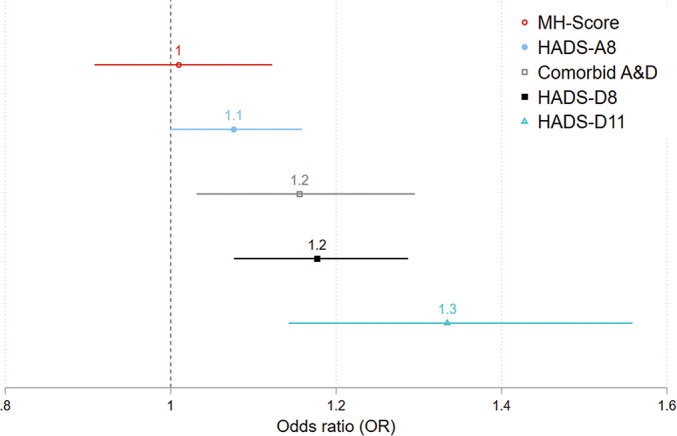
OR and 95% CI (95% CI) for poor mental health in rural areas (ref=urban areas).

In model 2, the indicator for urban–rural place of residence was added together with variables from model 1. Compared with urban residents, rural resident had an increased odds for reporting poor mental health on all outcomes except for mental distress. Figure 1 depicts ORs and 95% CIs from model 2.

Model 3 (table 2) expands model 2 by including an interaction term between the PRS and urban–rural living. In model 3, the main effect of the PRS for urban participants was 1.04 (95% CI 1.00 to 1.09) for HADS-D8 and 1.09 (95% CI 1.00 to 1.18) for HADS-D11, whereas the other main effects for urban participants were similar to the effects in model 1 for all participants. The interaction terms suggest a decreased risk for rural participants compared with urban participants associated with 1 SD increase in polygenic scores for moderate-to-severe depression (OR 0.96, 95% CI 0.89 to 1.03) and severe depression (OR 0.91, 95% CI 0.79 to 1.05), but these associations were not statistically significant. We found no evidence of interactions on the additive scale (online supplementary table 1). No interactions were found in models stratified either by sex or age (over/under 50 years).

**Figure 2:**
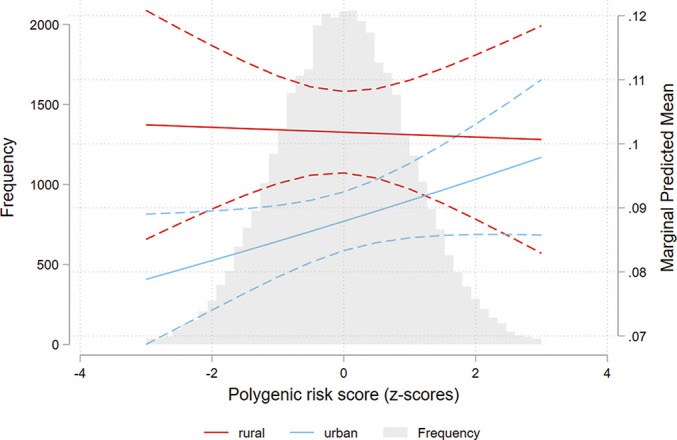
Predicted probability (95% CI) for having symptoms of depression (HADS-D8) by polygenic risk score and area characteristics (urban/rural). Distribution of frequencies according to PRS values in background. HADS, Hospital Anxiety and Depression Scale; PRS, polygenic risk score.

Figure 2 shows the predicted probability (95% CI) for moderate-to-severe symptoms of depression according to PRS and urbanicity and shows a different effect of the PRS for urban participants compared to rural participants. A test for simple slope for urban participants was not statistically significant (p=0.06).

Analyses with a three-group classification of urbanicity showed that there was a dose–response relationship with urbanicity, where the odds of reporting poor mental health increased with decreasing level of urbanicity (online supplementary table 2). No interactions were found between the PRS and urbanicity.

## DISCUSSION

Our results confirm prior findings suggesting that a PRS for depression has a small but significant association with the risk of mental health outcomes. However, we found no evidence that the effect of genetic propensity differs between urban and rural areas for any of the mental health outcomes examined.

### Comparison with previous research

Few previous studies have used a truly environmental spatial construct to investigate moderated effects of genetic propensity for mental health phenotypes. One study from the USA found that the genetic propensity for smoking predicted higher mean number of cigarettes smoked per day in neighbourhoods with a low level of social cohesion than in neighbourhoods with high social cohesion.^27^ A more recent study from the Netherlands tested interactions between a PRS for substance abuse and a number of neighbourhood characteristics and found that only 1 of 14 tested interactions was statistically significantly related to substance abuse.^28^ Another recent study suggests that a PRS for schizophrenia was more strongly related to treatment-resistant schizophrenia in rural and semiurban areas (HR: 1.20) compared with the capital area.^29^ Our study adds to the evidence of inconsistent findings in the GxE literature looking at higher-order environmental features. There may be methodological issues causing these inconsistencies or more fundamental flaws in the underlying theoretical models. Most studies have been rooted in the diathesis–stress framework, but the differential susceptibility model may also be important. However, variants from GWAS might not capture differential susceptibility and thus not constitute the best measure for GxE studies.^30^

### Interpretation of findings

The PRS we tested on five different symptoms of poor mental health was significantly associated with several of the mental health outcomes examined, but associations were relatively small. As a consequence, our ability to find GxE was small. While the GWAS found the reported genetic variants to be robust across three studies, they replicated poorly for the phenotypes in our sample (details available from the corresponding author). A possible explanation for this discrepancy is that the genetic variants used to calculate the PRS came from a GWAS on major depression,^21^ while the phenotypes we studied were symptoms of poor mental health.

Urbanicity may constitute a very heterogeneous environmental construct encompassing both risk factors and protective factors, for example, urban environments may be more stressful, but at the same time, access to health services or social networks may reduce stress and depression. Previous studies have largely studied environmental conditions that operate at the individual level, such as childhood trauma, SLE and social support.^12^ By contrast, a characteristic of the area where individuals reside capture higher-order effects that are more difficult to capture when using individual-level data, making it also more challenging to identify GxE interactions.

When studying gene-environment interactions (GxE), it is important to simultaneously check for gene-environment correlations (rGE), because what appears as interactions may in fact reflect clustering according to genetic propensities. While rGE reflect genetic differences in exposure to particular environments, GxE refers to *genetic differences in susceptibility* to particular environments.^31 32^ When testing rGE, we found the PRS predicted urban residence, thus suggesting gene-environment correlations. When interpreting this finding, it is possible that our suggestive gene-environment interaction for depression is in fact gene-environment correlation, that is, genetic propensity for depression is more prevalent in urban areas. A higher prevalence may occur when individuals self-select environments guided by their genetic predispositions. This makes the interpretation of GxE cumbersome, as the interaction might arise as a result of genetic propensities for urban residential choice. A closely related interpretation of this finding is that polygenic scores influence the risk of depression and anxiety earlier in life and that the latter influence the probability of residing in urban areas, reflecting ‘reverse causality’. While we have treated rGE as a disturbing element in the pursuit of GxE, it is an interesting phenomenon largely ignored in the GxE literature, but it might be equally or even more important in the aetiology of mental health problems.

Our study has several strengths. It is conducted in a large general population sample and we used validated instruments as outcomes. Urbanicity, constructed from an external data source, was based on a detailed classification of place of residence in accordance with Statistics Norway’s definition of urban areas. Delineating urban–rural neighbourhoods based on wards is preferable, because this is the lowest spatial scale possible and corresponds closely with neighbourhoods, thus making them sociodemographic homogenous within and heterogenous between. We developed a PRS based on the most recent GWAS reporting 102 genome-wide significant associations with major depression in populations of European ancestry.^21^ Thus, we had a very large and independent discovery sample that allowed us to derive the PRS.^9^

Nevertheless, a number of limitations should be considered in this study. The response rate was 54% and a non-participation study has shown that non-participants had poorer health.^16^ Missing was in general low (<5%), but the MHI index with 7.4% missingness can be biased. The symptom scores used as outcomes were collected at one timepoint only. The genetic variants used to calculate the PRS were derived from a GWAS on major depression, and while the phenotypes we have studied are closely related to major depression, they are nevertheless symptoms and not clinically assessed diagnoses. Further, we lacked the possibility to adjust analyses for genotyping arrays. Finally, we performed an analysis on participants with valid information and made no attempt to impute missing data.

## CONCLUSION

The PRS had a significant but small association with symptoms of anxiety, comorbid anxiety and depression and mental distress. We found no support for a differential effect of genetic propensity between urban and rural neighbourhoods. While our findings do not support the hypothesis of gene-environment interactions using PRS, other approaches such as genome-wide by environment interaction studies represents a potential alternative to understand how genetic variants interact with specific features of the urban environment.^33^ The value of doing GxE studies ultimately lies in their potential for advancing our understanding of causal pathways with respect to both genetic and environmental mechanisms in the origin of adverse mental health.

**What is already known on this topic**

- Studies suggest that genetic factors play an important role in both anxiety and depression and that genetic propensity may be contingent on environmental characteristics, that is, environment may modify the effect of genetic propensity.

**What this study adds**

- Genetic propensity for major depression, operationalised through a polygenic risk score, was associated with symptoms of anxiety, depression and mental distress, but there was no evidence of modification by residential urbanicity.
