# Supplementary material for: Does urbanicity modify the relationship between a polygenic risk score for depression and mental health symptoms? Cross-sectional evidence from the observational HUNT Study in Norway
Source: J Epidemiol Community Health. 2020 Jun 24;75(5):420–5. doi: 10.1136/jech-2020-214256 (PMC8053322; doi:10.1136/jech-2020-214256)
Supplement: Supplementary data [file jech-2020-214256supp001.pdf]

Supplementary material

Genotyping and SNP imputation procedures in HUNT

The genotype quality control and imputation has been conducted by the K.G. Jebsen Center for Genetic Epidemiology, Department of public health and nursing, Faculty of medicine and health sciences, Norwegian University of Science and Technology (NTNU). The quality control procedures are outlined in a fact sheet provided by the KG Jebsen Center for Genetic Epidemiology (1). The following information is quoted from this fact sheet.

"In total, DNA from 71,860 HUNT samples was genotyped using one of three different Illumina HumanCoreExome arrays (HumanCoreExome12 v1.0, HumanCoreExome12 v1.1 and UM HUNT Biobank v1.0). Samples that failed to reach a 99% call rate, had contamination > 2.5% as estimated with BAF Regress (Jun et al. , 2012), large chromosomal copy number variants, lower call rate of a technical duplicate pair and twins, gonosomal constellations other than XX and XY, or whose inferred sex contradicted the reported gender, were excluded. Samples that passed quality control were analysed in a second round of genotype calling following the Genome Studio quality control protocol".

"Variants were excluded if (1) their probe sequences could not be perfectly mapped to the reference genome, cluster separation was < 0.3, Gentrain score was < 0.15, showed deviations from Hardy Weinberg equilibrium in unrelated samples of European ancestry with p-value < 0.0001), their call rate was < 99%, or another assay with higher call rate genotyped the same variant. "

"Imputation was performed on the 69,716 samples of recent European ancestry using Minimac3 (v2.0.1,http://genome.sph.umich.edu/wiki/Minimac3) (Das et al. , 2016) with default settings (2.5 Mb reference based chunking with 500kb windows) and a customized Haplotype Reference consortium release 1.1 (HRC v1.1) for autosomal variants and HRC v1.1 for chromosome X variants (McCarthy et al. , 2016). The customized reference panel represented the merged panel of two reciprocally imputed reference panels: (1) 2,201 low-coverage whole-genome sequences samples from the HUNT study and (2) HRC v1.1 with 1,023 HUNT WGS samples removed before merging." Imputed variants with Rsq < 0.3 were excluded (1).

References

1. K.G. Jebsen Center for Genetic Epidemiology. All-in fast track SNP's. 2018

Phenotypes for major depression used in Howard et. al. (2019)

UK biobank:

"Have you ever seen a general practitioner for nerves, anxiety, tension or depression?"

"Have you ever seen a psychiatrist for nerves, anxiety, tension or depression?"

23 and me:

Individuals that self-reported as having received a clinical diagnosis or treatment for depression

PGC (Major depressive disorder working group of the Psychiatric Genomics Consortium):

Clinically-derived phenotypes for major depressive disorder (MDD)

Supplementary table 1. Associations between a polygenic risk score for depression and 5 mental health outcomes. Beta coefficients and 95 percent confidence intervals (95% CI) reported in base 10 logarithms

	HADS-D (>=8)		HADS-D (>=11)		HADS-A (>=8)	
	coef.	95% CI	coef.	95% CI	coef.	95% CI
PRS (z-score)	$3.24x10^{-3}$	($-2.56x10^{-4}$ to $6.74x10^{-3}$)	$1.63x10^{-3}$	($-1.35x10^{-4}$ to $3.40x10^{-3}$)	**$9.27x10^{-3}$**	**($5.10x10^{-3}$ to $1.34x10^{-2}$)**
Sex						
Females	0	ref.	0	ref.	0	ref.
Males	**$1.52x10^{-2}$**	**($9.23x10^{-3}$ to $2.11x10^{-2}$)**	$2.68x10^{-3}$	($-3.20x10^{-4}$ to $5.68x10^{-3}$)	**$-6.88x10^{-2}$**	**($-7.58x10^{-2}$ to $-6.17x10^{-2}$)**
Urbanicity						
Urban	0	ref.	0	ref.	0	ref.
Rural	**$1.41x10^{-2}$**	**($6.41x10^{-3}$ to $2.18x10^{-2}$)**	**$6.48x10^{-3}$**	**($2.98x10^{-3}$ to $9.98x10^{-3}$)**	$8.82x10^{-3}$	($-5.63x10^{-5}$ to $1.77x10^{-2}$)
PRS*Rural	$-3.61x10^{-3}$	($-1.01x10^{-2}$ to $2.85x10^{-3}$)	$-1.96x10^{-3}$	($-5.22x10^{-3}$ to $1.30x10^{-3}$)	$8.80x10^{-4}$	($-6.81x10^{-3}$ to $8.57x10^{-3}$)

	Comorbid A&D		Mental health score	
	coef.	95% CI	coef.	95% CI
PRS (z-score)	$2.25x10^{-3}$	($-2.69x10^{-4}$ to $4.77x10^{-3}$)	**$5.04x10^{-3}$**	**($1.93x10^{-3}$ to $8.14x10^{-3}$)**
Sex				
Females	0	ref.	0	ref.
Males	**$-1.12x10^{-2}$**	**($-1.55x10^{-2}$ to $-6.93x10^{-3}$)**	**$-1.33x10^{-2}$**	**($-1.86x10^{-2}$ to $-8.03x10^{-3}$)**
Urbanicity				
Urban	0	ref.	0	ref.
Rural	**$6.59x10^{-3}$**	**($1.43x10^{-3}$ to $1.17x10^{-2}$)**	$4.99x10^{-4}$	($-6.36x10^{-3}$ to $7.36x10^{-3}$)
PRS*Rural	$-6.28x10^{-4}$	($-5.27x10^{-3}$ to $4.02x10^{-3}$)	$-7.46x10^{-4}$	($-6.50x10^{-3}$ to $5.01x10^{-3}$)

*Adjusted for age (restricted cubic splines with 4 knots) and first 5 principal components. Estimates in **bold** significant at $p<0.05$

Supplementary table 2. Associations between a polygenic risk score for depression and 5 mental health outcomes. Odds ratio (OR) and 95 percent confidence interval (95% CI).

	HADS-D (>=8)		HADS-D (>=11)		HADS-A (>=8)		Comorbid A&D		Mental health score	
	OR	95% CI	OR	95% CI	OR	95% CI	OR	95% CI	OR	95% CI
Model 1										
Fixed effects										
PRS (z-score)	1.03	(0.99 to 1.06)	1.05	(0.98 to 1.12)	**1.08**	**(1.05 to 1.12)**	**1.05**	**(1.00 to 1.10)**	**1.08**	**(1.04 to 1.12)**
Sex										
Females	1	ref.	1	ref.	1	ref.	1	ref.	1	ref.
Males	**1.20**	**(1.12 to 1.28)**	1.14	(0.99 to 1.30)	**0.55**	**(0.52 to 0.59)**	**0.78**	**(0.70 to 0.86)**	**0.81**	**(0.75 to 0.88)**
Random effects										
Variance (se)	0.070	(0.015)	0.097	(0.039)	0.036	(0.009)	0.068	(0.022)	0.081	(0.019)
Model 2										
Fixed effects										
PRS (z-score)	1.03	(0.99 to 1.06)	1.05	(0.98 to 1.12)	**1.08**	**(1.05 to 1.12)**	**1.05**	**(1.00 to 1.10)**	**1.08**	**(1.04 to 1.12)**
Sex										
Females	1	ref.	1	ref.	1	ref.	1	ref.	1	ref.
Males	**1.19**	**(1.11 to 1.28)**	1.13	(0.98 to 1.30)	**0.55**	**(0.52 to 0.59)**	**0.77**	**(0.70 to 0.85)**	**0.81**	**(0.75 to 0.88)**
Urbanicity										
Urban	1	ref.	1	ref.	1	ref.	1	ref.	1	ref.
Less urban	**1.13**	**(1.01 to 1.27)**	1.06	(0.87 to 1.30)	**1.10**	**(1.00 to 1.21)**	1.05	(0.91 to 1.21)	1.07	(0.94 to 1.23)
Rural	**1.28**	**(1.13 to 1.43)**	**1.38**	**(1.14 to 1.69)**	**1.15**	**(1.04 to 1.26)**	**1.19**	**(1.03 to 1.39)**	1.06	(0.92 to 1.21)
Random effects										
Variance (se)	0.057	(0.014)	0.081	(0.035)	0.032	(0.009)	0.060	(0.022)	0.080	(0.019)
Model 3										
Fixed effects										
PRS (z-score)	1.03	(0.97 to 1.10)	1.07	(0.94 to 1.22)	**1.11**	**(1.05 to 1.17)**	1.06	(0.97 to 1.15)	**1.14**	**(1.05 to 1.22)**
Sex										
Females	1	ref.	1	ref.	1	ref.	1	ref.	1	ref.
Males	**1.19**	**(1.11 to 1.28)**	1.13	(0.99 to 1.30)	**0.55**	**(0.52 to 0.59)**	**0.77**	**(0.70 to 0.85)**	**0.81**	**(0.75 to 0.88)**
Urbanicity										
Urban	1	ref.	1	ref.	1	ref.	1	ref.	1	ref.
Less urban	**1.13**	**(1.00 to 1.27)**	1.06	(0.87 to 1.29)	**1.11**	**(1.01 to 1.21)**	1.05	(0.91 to 1.21)	1.08	(0.94 to 1.24)
Rural	**1.28**	**(1.13 to 1.43)**	**1.39**	**(1.14 to 1.69)**	**1.15**	**(1.04 to 1.26)**	**1.19**	**(1.03 to 1.40)**	1.06	(0.93 to 1.22)
PRS*Urbanicity										
Less urban	1.02	(0.93 to 1.11)	1.02	(0.86 to 1.22)	0.97	(0.90 to 1.04)	1.00	(0.89 to 1.12)	0.92	(0.83 to 1.01)
Rural	0.97	(0.88 to 1.06)	0.92	(0.77 to 1.10)	0.98	(0.91 to 1.06)	0.98	(0.87 to 1.11)	0.94	(0.85 to 1.05)
Random effects										
Variance (se)	0.057	(0.014)	0.081	(0.035)	0.032	(0.009)	0.060	(0.022)	0.080	(0.019)

*Adjusted for age (restricted cubic splines with 4 knots) and first 5 principal components. Estimates in **bold** significant at $p<0.05$
